# Comparative multi-target computational profiling of ebelin lactone across cognition-associated proteins

**DOI:** 10.3389/fbinf.2026.1952759

**Published:** 2026-10-23

**Authors:** Monica Lokanadham, Ramalingam Chidambaram

**Affiliations:** School of Bio Sciences and Technology, Vellore Institute of Technology, Vellore, India

**Keywords:** bacopa monnieri, cognition, ebelin lactone, MM/GBSA, molecular docking, molecular dynamics, mortalin, neuritin

## Abstract

**Background:**

Ebelin lactone (EL) is a bacoside-related triterpenoid derivative produced through acid hydrolysis and has previously shown binding affinity toward the M1 muscarinic and 5-HT2A receptors. However, its structural compatibility with proteins involved in synaptic plasticity, neuronal proteostasis, mitochondrial stress responses, and neurite remodeling has not been systematically evaluated.

**Methods:**

We evaluated EL against proteins associated with cognition: Alpha-actinin-4 (ACTN4), Brain-derived neurotrophic factor (BDNF), Complexin-1 (CPLX1), Heat shock protein 72 (HSP70-1), Heat shock protein family member 9 (HSPA9), and Neuritin (NRN1). The workflow integrated ADMET-AI predictions, blind docking using GNINA with automated search space generation and Vina-based pose scoring, 100-ns GROMACS molecular dynamics for five selected complexes and their corresponding apo forms, trajectory analyses, principal component-based free-energy landscape analysis, and MM/GBSA binding-energy estimation with per-residue energy decomposition.

**Results:**

HSPA9 showed the most favorable docking score of (−9.13) kcal mol^−1^, followed by ACTN4 (−8.65), BDNF (−8.57), HSP70-1 (−8.19), NRN1 (−7.87), and CPLX1 (−7.00). CPLX1 was not advanced to MD simulations because it had a small, predicted pocket and the least favorable score. Throughout the 100-ns MD simulations, the ACTN4 fragment had the lowest mean protein RMSD (0.163 ± 0.021 nm). In contrast, the full precursor model of BDNF showed the highest mean protein RMSD of 2.125 ± 0.388 nm. Comparison with the corresponding apo trajectories showed target-dependent changes, with NRN1–EL exhibiting lower mean RMSD and RMSF than apo NRN1, although its Rg and SASA values were higher. NRN1 exhibited the highest mean protein–ligand hydrogen-bond count (0.575 ± 0.501) and the most negative MM/GBSA binding-energy estimate (−39.21 ± 3.24 kcal mol^−1^), followed by HSPA9 (−37.93 ± 2.60) and BDNF (−33.83 ± 3.49). ADMET results indicated high lipophilicity (logP 7.018), low aqueous solubility, and mixed permeability-related signals.

**Conclusion:**

Based on the integrated findings, NRN1 emerged as the overall most promising computational candidate for experimental follow-up. These findings require experimental validation.

## Graphical Abstract

**Figure:**
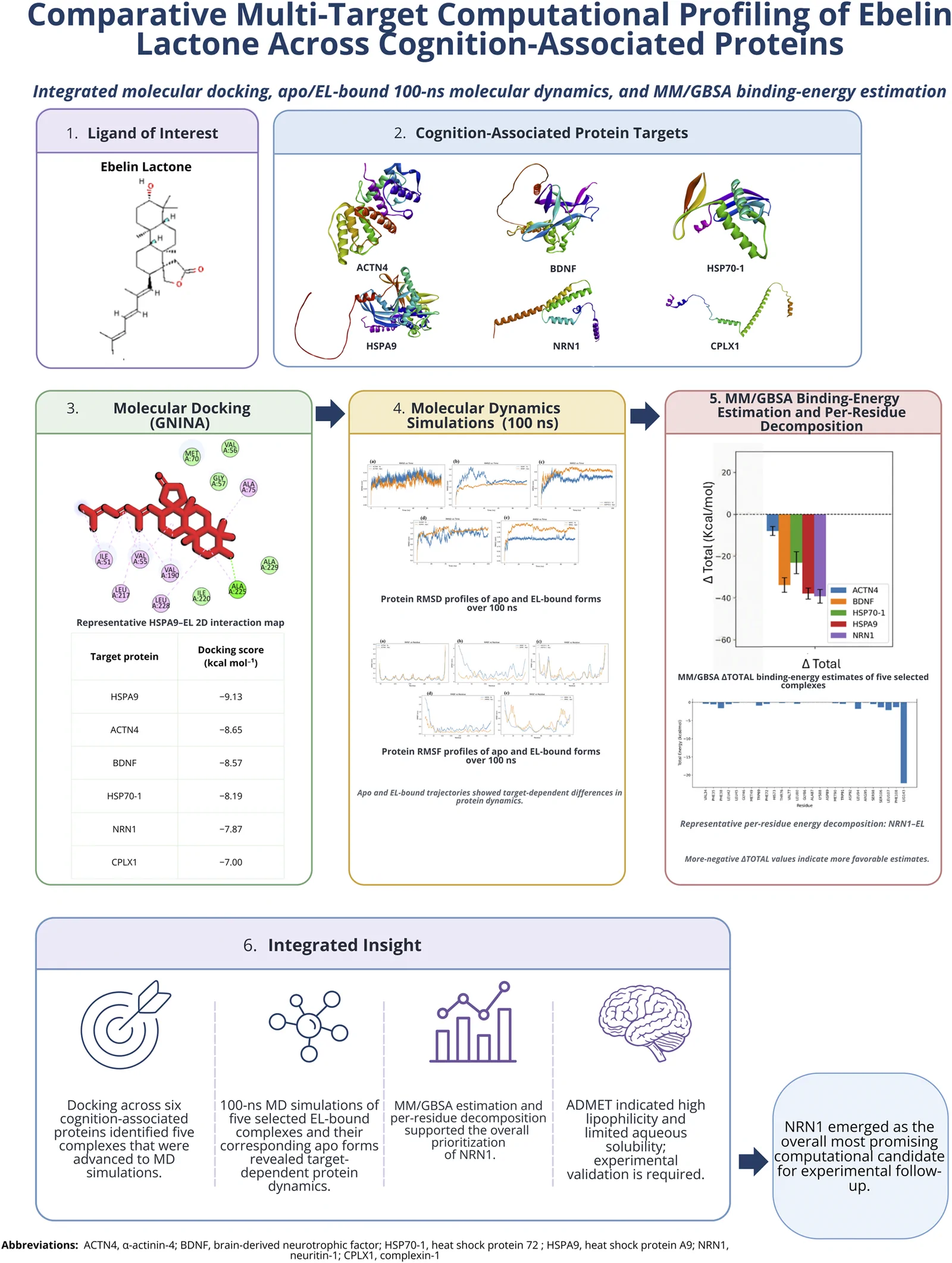
Created by using Canva Pro.

## 1 Introduction

Cognitive impairment plays a key role in many neurodegenerative and neuropsychiatric disorders. It is strongly associated with disturbances in synaptic plasticity, neurite structure, mitochondrial balance, and the body’s protein-quality-control mechanisms. In 2021, dementia impacted about 57 million people globally, with nearly 10 million new cases diagnosed each year (World Health Organization, 2026). Even though there have been significant breakthroughs in understanding disease biology, effective ways to protect or restore cognitive function are still scarce. As a result, compounds capable of modulating complementary cellular processes continue to attract interest.

*Bacopa monnieri (L*.*) Wettst* is a medicinal plant from the Plantaginaceae family widespread across tropical and subtropical regions, including India. *Bacopa monnieri* has a longstanding history of use for enhancing memory and cognitive function. Systematic reviews and meta-analyses of randomized trials have reported potential advantages for selected memory and attention metrics; however, heterogeneity of studies and variation in extracts, dosages, and duration limit broad conclusions (Pase et al., 2012; Kongkeaw et al., 2014). Bacoside A is a mixture of dammarane-type triterpenoid saponins. Its deglycosylation yields jujubogenin and pseudojujubogenin-related aglycones, whereas acid hydrolysis generates ebelin lactone (EL) and bacogenin A1 (Ramasamy et al., 2015). Compared with the parent bacosides, EL has fewer hydrogen-bonding groups and a smaller polar surface, properties that may improve membrane partitioning while potentially elevating hydrophobicity and reducing aqueous solubility. Evidence pertaining specifically to EL remains limited. A foundational computational and receptor-binding study reported micromolar binding affinities of EL for the M1 muscarinic and 5-HT2A receptors, along with more favorable predicted CNS drug-like properties than those of the parent glycosides (Ramasamy et al., 2015). A recent review also discussed EL among the bacopa-derived compounds, which have potential relevance to the neurological disorders (Vinod et al., 2022). However, the potential interactions of EL with cognition-relevant proteins involved in neuronal structure, synaptic function, and cellular resilience have not been explored in detail.

The selected targets collectively represent complementary cognition-associated processes rather than a single canonical pathway. Selection was based on published evidence linking them to cognitive resilience, synaptic plasticity or function, proteostasis, mitochondrial stress responses, or neurite development, together with the availability of a usable human structural input. Proteome-wide studies have linked ACTN4, CPLX1, and NRN1 to the preservation of cognitive function in elderly individuals (Yu et al., 2020). BDNF signaling plays an important role in dendritic-spine development and structural synaptic plasticity (Zagrebelsky et al., 2020). CPLX1 is a part of presynaptic release machinery. Changes in complexin-1 levels have been observed in the hippocampal tissue of individuals with Alzheimer’s disease (Haytural et al., 2021). A comprehensive proteomic analysis of the presynaptic soluble N-ethylmaleimide-sensitive factor attachment protein receptor (SNARE) interaction network also linked specific synaptic proteoforms to cognitive reserve in older adults (Ramos-Miguel et al., 2021).

HSP70-1, also known as heat shock protein 72, is a stress-responsive molecular chaperone involved in ATP-dependent protein folding and proteostasis. Hsp70-mediated protein-quality-control pathways are important for maintaining cellular homeostasis and limiting protein misfolding associated with neurodegenerative diseases (Mylvaganam et al., 2021; Ghosh et al., 2024). HSPA9, also known as mortalin or GRP75, is a mitochondrial chaperone involved in mitochondrial protein import, protein folding, and cellular stress responses (Flachbartová and Kovacech, 2013). Differential expression of mortalin isoforms has been observed in hippocampal tissue from individuals with Alzheimer’s disease, with the expression pattern influenced by APOE genotype (Osorio et al., 2007). Human proteomic studies have associated NRN1 with cognitive resilience in Alzheimer’s disease, while emerging evidence suggests that it contributes to neuronal plasticity and structural remodeling (Hurst et al., 2023; Agrawal et al., 2026).

To the best of our knowledge, EL has not previously been evaluated across this group of proteins involved in structural plasticity, synaptic function, proteostasis, mitochondrial homeostasis, and neurite development within a unified docking and molecular dynamics workflow. Thus, we compared its predicted behavior on the selected targets. The analysis included *in silico* evaluation of the ADMET properties of EL and blind docking against six proteins. Then, 100-ns molecular dynamics simulations were performed on five protein–EL complexes and their corresponding apo forms. The simulated complexes were further evaluated by considering the simulation trajectories, free-energy landscape analysis, MM/GBSA binding-energy estimation, and per-residue energy decomposition.

## 2 Materials and methods

### 2.1 Study design

The comparative *in silico* study was carried out sequentially using a workflow encompassing ligand retrieval and preparation, ADMET prediction, protein structure selection and preparation, molecular docking, molecular dynamics simulations, trajectory analysis, principal component analysis (PCA), free-energy landscape (FEL) analysis, MM/GBSA binding-energy estimation, and per-residue energy decomposition analysis. The selected proteins differed in size, fold, structural coverage, and binding-site characteristics. Therefore, cross-target comparisons were used for relative prioritization within the current workflow, not as a direct measure of pharmacological affinity.

### 2.2 Ligand retrieval and preparation

The ligand, Ebelin lactone (CID: 15559069), was obtained from the PubChem database as a structure data file and prepared using a predefined ScientiFlow workflow^1^. Open Babel was used for ligand preparation and energy minimization with the MMFF95 force field for 1,000 steps (O’Boyle et al., 2011). The ligand structure was exported in PDB format. The SMILES annotation of EL was used as an input for ADMET prediction prior to molecular docking.

### 2.3 ADMET prediction

The ADMET properties of EL were predicted using the ADMET-AI module, an inbuilt module in ScientiFlow. The SMILES annotation of EL was used as input. ADMET-AI generated predictions for physicochemical properties as well as absorption, distribution, metabolism, excretion, and toxicity endpoints (Swanson et al., 2024). The complete set of prediction outputs is provided in Supplementary Table S1.

### 2.4 Target selection and protein preparation

The analysis focused on six human cognition-associated proteins: ACTN4, BDNF, CPLX1, HSP70-1, HSPA9, and NRN1. These targets were selected through a literature-guided review based on published evidence linking them to cognitive resilience, synaptic plasticity or function, proteostasis, mitochondrial stress responses, or neurite development. UniProt was used to confirm protein nomenclature and reference sequence information, whereas structural coordinates were obtained from the RCSB Protein Data Bank or the AlphaFold Protein Structure Database (Burley et al., 2019; Varadi et al., 2022; Bateman et al., 2023).

A full-length experimentally determined structure of human ACTN4 was not available. Therefore, PDB 2R0O was used as the ACTN4 structural input. This experimentally resolved structure represents a 237-residue K255E actin-binding domain fragment determined at a resolution of 2.20 Å. AlphaFold models were used for the remaining targets because suitable experimental structures corresponding to the selected structural inputs were unavailable. The BDNF input represented the precursor protein. HSP70-1 was represented by the 151-residue AF-Q9UQC1-F1 model corresponding to UniProt accession Q9UQC1 and annotated as heat shock protein 72. The HSPA9 structural input comprised residues 1–380 of its 679-residue reference sequence. The computational findings were therefore interpreted at the level of the specific structural inputs used in the present study. Details of the structural inputs and reference sequence lengths are summarized in Table 1.

**TABLE 1:** Structural inputs used for molecular docking and molecular dynamics simulations.

Target	Name	Reference sequence length (aa)	UniProt ID	Code	Method
ACTN4	Alpha-actinin-4	911	O43707	PDB ID: 2R0O	X-ray, 2.20 Å
BDNF	Neurotrophic factor BDNF precursor form	247	P23560	AF-P23560-F1	Predicted
CPLX1	Complexin-1	134	O14810	AF-O14810-F1	Predicted
HSP70-1	Heat shock protein 72	151	Q9UQC1	AF-Q9UQC1-F1	Predicted
HSPA9 (mortalin)	Stress-70 protein, mitochondrial	679	P38646	AF-P38646-F1	Predicted
NRN1	Neuritin	142	Q9NPD7	AF-Q9NPD7-F1	Predicted

The protein structures were prepared using the ScientiFlow workflow. Water molecules and heteroatoms were removed, after which polar hydrogen atoms were added and Kollman charges were assigned. The prepared protein structures were saved in PDB format for subsequent molecular docking.

### 2.5 Molecular docking

GNINA was used through the predefined ScientiFlow workflow for molecular docking (McNutt et al., 2021). Blind docking was performed for all six proteins because experimentally validated binding sites were not uniformly available across the selected targets, most of which were represented by AlphaFold models. EL was used as the input ligand and docked independently against each target. The docking search space was generated automatically by GNINA, and the resulting poses were ranked using the Vina scoring function. Ligand normalization and format conversion were performed using RDKit (Landrum, 2006) and gypsum_dl (Ropp et al., 2019). Docking was carried out with a flexibility setting of 8, yielding 9 binding modes for each ligand. All protein–ligand complexes were examined through post-docking analysis, with emphasis on the docking scores and the types of interactions observed. Based on the docking results, the ACTN4–EL, BDNF–EL, HSP70-1–EL, HSPA9–EL, and NRN1–EL complexes were selected for molecular dynamics simulations to examine their behavior under dynamic conditions. CPLX1–EL was not advanced to molecular dynamics because it had the least favorable docking score and occupied a comparatively small, predicted binding pocket.

### 2.6 Molecular dynamics simulations

The selected protein–ligand complexes and their corresponding apo forms were considered for molecular dynamics simulations using the automated system developed in ScientiFlow. Simulations were performed using GROMACS 2024.3 (Abraham et al., 2015). The protein and ligand coordinates were extracted into separate PDB files and uploaded to ScientiFlow for simulation preparation. The CHARMM36 force field was used for protein parameterization (Huang and MacKerell, 2013), while the EL topology was generated using SwissParam within the ScientiFlow workflow (Bugnon et al., 2023). The simulation setup involved placing the protein–ligand complex into the dodecahedral periodic box. The system was solvated with the use of the TIP3P water model, followed by the addition of sodium and chloride ions to neutralize the entire system and obtain a physiological salt concentration of 0.15 M NaCl. The energy minimization was conducted using the steepest descent method to eliminate steric clashes and produce a reliable initial structure. System equilibration was carried out in two stages. Initially, the NVT ensemble was applied at 300 K with position restraints on the heavy atoms. This was followed by NPT equilibration at a pressure of 1 bar. After equilibration, the production MD simulation was performed for 100 ns using a time step of 2 fs under periodic boundary conditions. Long-range electrostatic interactions were handled using the particle-mesh Ewald method.

### 2.7 Trajectory and binding-energy analyses

Trajectory analysis was carried out to assess the stability of apo forms and corresponding protein–ligand complexes over the course of the simulations. The analysis included root-mean-square deviation (RMSD), root-mean-square fluctuation (RMSF), radius of gyration (Rg), hydrogen-bond formation, and solvent-accessible surface area (SASA) (Eisenhaber et al., 1995). Mean and standard deviation values were calculated across the trajectory frames and were treated as descriptive statistics rather than independent biological or technical replicates. Principal component analysis (PCA) was performed using protein conformational coordinates, and free-energy landscape (FEL) maps were generated along the first two principal components to examine the conformational states sampled during the simulations.

Binding-energy estimation and per-residue energy decomposition were performed using gmx_MMPBSA over frames 9,000–10,000 of the trajectories, corresponding to 1,001 frames (Valdés-Tresanco et al., 2021). The calculations used the Generalized Born OBC2 model (igb = 5) with a salt concentration of 0.150 M. The reported components included van der Waals forces, electrostatic interactions, polar and nonpolar solvation effects, and the overall MM/GBSA energy values. Entropic contributions were not included.

## 3 Results

### 3.1 ADMET predictions

The SMILES annotation of ebelin lactone (EL), shown in Figure 1G, was used for ADMET prediction, and the complete results are provided in Supplementary Table S1. EL had a molecular weight of 454.695 Da, a calculated logP of 7.0181, three hydrogen-bond acceptors, one hydrogen-bond donor, a topological polar surface area of 46.53 Å^2^, and a quantitative estimate of drug-likeness of 0.36811. Among the absorption- and permeability-related endpoints, the HIA Hou model, which evaluates human intestinal absorption, produced an output of 0.992545, while the PAMPA NCATS model, which evaluates passive membrane permeability, produced an output of 0.925511. The Bioavailability Ma model, which evaluates oral bioavailability, produced an output of 0.44261. The P-glycoprotein Broccatelli model, which evaluates P-glycoprotein inhibition, produced an output of 0.821793, while the BBB Martins model, which evaluates blood–brain barrier penetration, produced an output of 0.206425. The Caco-2 Wang model, a regression endpoint for intestinal permeability, produced an output of −5.23412. Because the generated ADMET-AI output did not provide endpoint-specific decision thresholds, class definitions, or prediction confidence information, these values were retained as model-generated predictions and were not used to make definitive claims regarding intestinal absorption, oral bioavailability, or CNS exposure. The output also included predictions for AMES mutagenicity, carcinogenicity, ClinTox, drug-induced liver injury, hERG inhibition, and stress-response endpoints. These toxicity-related outputs were likewise retained as model-generated predictions rather than interpreted as definitive positive or negative findings. EL had a predicted solubility output of −6.79951 together with a calculated logP of 7.0181, indicating high predicted lipophilicity and limited predicted aqueous solubility. These properties may create challenges for formulation and bioavailability; therefore, formulation development or structural optimization may be considered to improve the solubility of EL.

**FIGURE 1:**
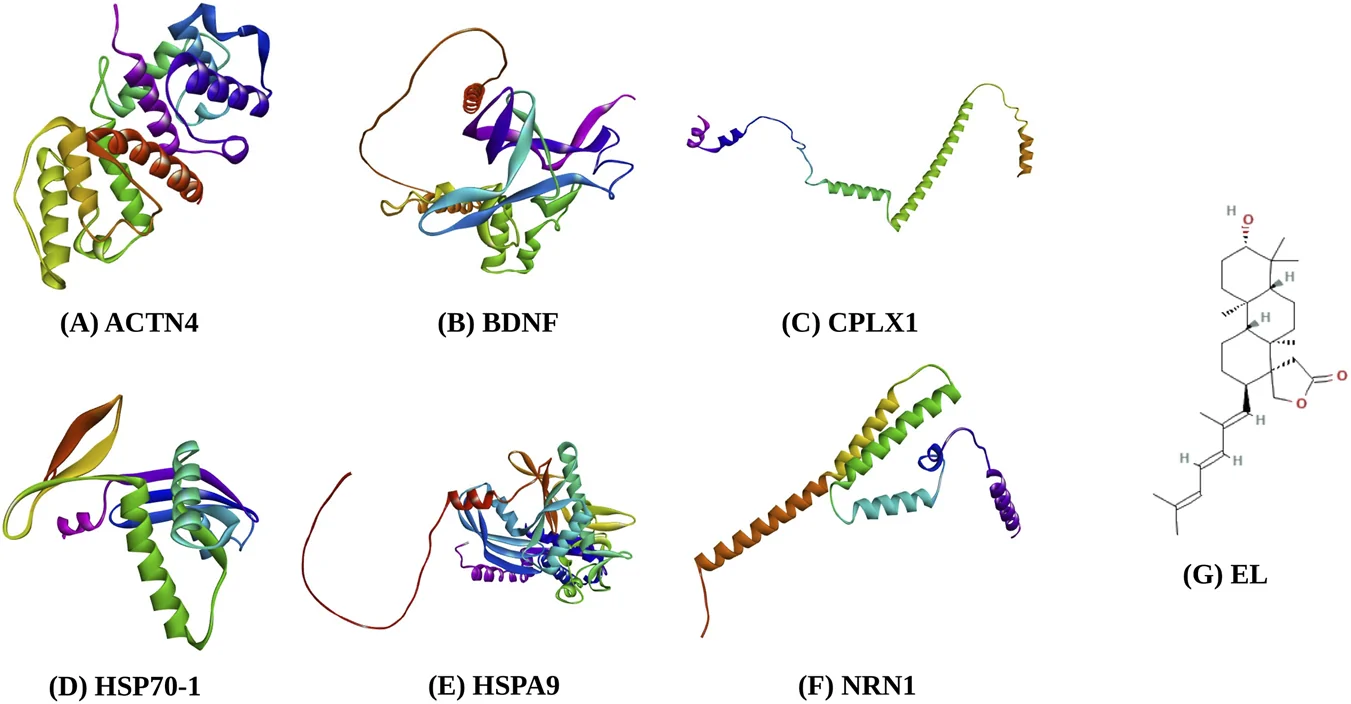
Three-dimensional structures of the selected proteins: **(A)** ACTN4, **(B)** BDNF, **(C)** CPLX1, **(D)** HSP70-1, **(E)** HSPA9, and **(F)** NRN1; and **(G)** the two-dimensional chemical structure of ebelin lactone (EL).

### 3.2 Structural inputs of the selected targets

Six cognition-associated proteins were included in the study: ACTN4, BDNF, CPLX1, HSP70-1, HSPA9, and NRN1. Their structural inputs are summarized in Table 1, while the corresponding 3D protein structures and the 2D structure of EL are presented in Figure 1. ACTN4 was represented by the experimentally determined PDB structure 2R0O, whereas AlphaFold-predicted models were used for BDNF, CPLX1, HSP70-1, HSPA9, and NRN1. The selected structural inputs differed in sequence coverage and structural source, as described in Section 2.4, and these differences were considered when interpreting the docking and molecular dynamics results.

### 3.3 Molecular docking and interaction profiles

EL was docked against ACTN4, BDNF, CPLX1, HSP70-1, HSPA9, and NRN1. The ScientiFlow output for each complex was examined for the Vina score and predicted residue contacts, including conventional hydrogen bonds. The docking scores ranged from −9.13 to −7.00 kcal mol^−1^, as summarized in Table 2.

**TABLE 2:** Docking scores and predicted residue contacts in the selected EL poses.

Complex	Docking score (kcal mol^−1^)	Conventional H-bond	van der waals contacts	Hydrophobic/π-associated contacts
ACTN4–EL	−8.65	None	GLN52, GLU255, ASP253, THR249	PHE46, TRP48, ALA250, ARG251
BDNF–EL	−8.57	None	PHE63, ILE67, GLU73, ASP74, GLU112	LEU70, LEU71, LEU109, LEU110, TYR113
CPLX1–EL	−7.00	LYS49	GLU46, ALA53, GLU56	ALA50, TYR52
HSP70-1–EL	−8.19	None	Not reported	PHE6, VAL17, VAL19, TYR21, PHE28, ILE33, MET36
HSPA9–EL	−9.13	ALA225	ILE220, VAL56, GLY57, MET70, ALA229	ILE51, VAL55, ALA75, VAL190, LEU217, LEU228
NRN1–EL	−7.87	None	PHE38, LEU42, LEU45, PHE72, ALA87, SER98, PHE108	TRP69, LEU80, MET90, TRP91, LEU94, LEU107

Residue contacts were assigned from the two-dimensional interaction maps. Docking scores are Vina scores obtained from the GNINA blind-docking workflow.

The ACTN4–EL complex had a docking score of −8.65 kcal mol^−1^. In the selected pose, no conventional hydrogen bond was identified. Van der Waals contacts involved GLN52, GLU255, ASP253, and THR249, while hydrophobic or π-associated contacts involved PHE46, TRP48, ALA250, and ARG251. The BDNF–EL complex had a docking score of −8.57 kcal mol^−1^, and no conventional hydrogen bond was identified. Van der Waals contacts involved PHE63, ILE67, GLU73, ASP74, and GLU112, while hydrophobic or π-associated contacts involved LEU70, LEU71, LEU109, LEU110, and TYR113.

The CPLX1–EL complex had the least favorable docking score of −7.00 kcal mol^−1^. One conventional hydrogen bond was identified with LYS49. Van der Waals contacts involved GLU46, ALA53, and GLU56, while hydrophobic or π-associated contacts involved ALA50 and TYR52. EL occupied a comparatively small, predicted binding pocket in CPLX1. The HSP70-1–EL complex had a docking score of −8.19 kcal mol^−1^. No conventional hydrogen bond was identified, and the selected pose was characterized mainly by hydrophobic or π-associated contacts involving PHE6, VAL17, VAL19, TYR21, PHE28, ILE33, and MET36.

The HSPA9–EL complex had the most favorable docking score of −9.13 kcal mol^−1^. One conventional hydrogen bond was identified with ALA225. Van der Waals contacts involved VAL56, GLY57, MET70, ILE220, and ALA229, while hydrophobic or π-associated contacts involved ILE51, VAL55, ALA75, VAL190, LEU217, and LEU228. The NRN1–EL complex had a docking score of −7.87 kcal mol^−1^, and no conventional hydrogen bond was identified. Van der Waals contacts involved PHE38, LEU42, LEU45, PHE72, ALA87, SER98, and PHE108, while hydrophobic or π-associated contacts involved TRP69, LEU80, MET90, TRP91, LEU94, and LEU107. The 2D interaction maps are presented in Figure 2.

**FIGURE 2:**
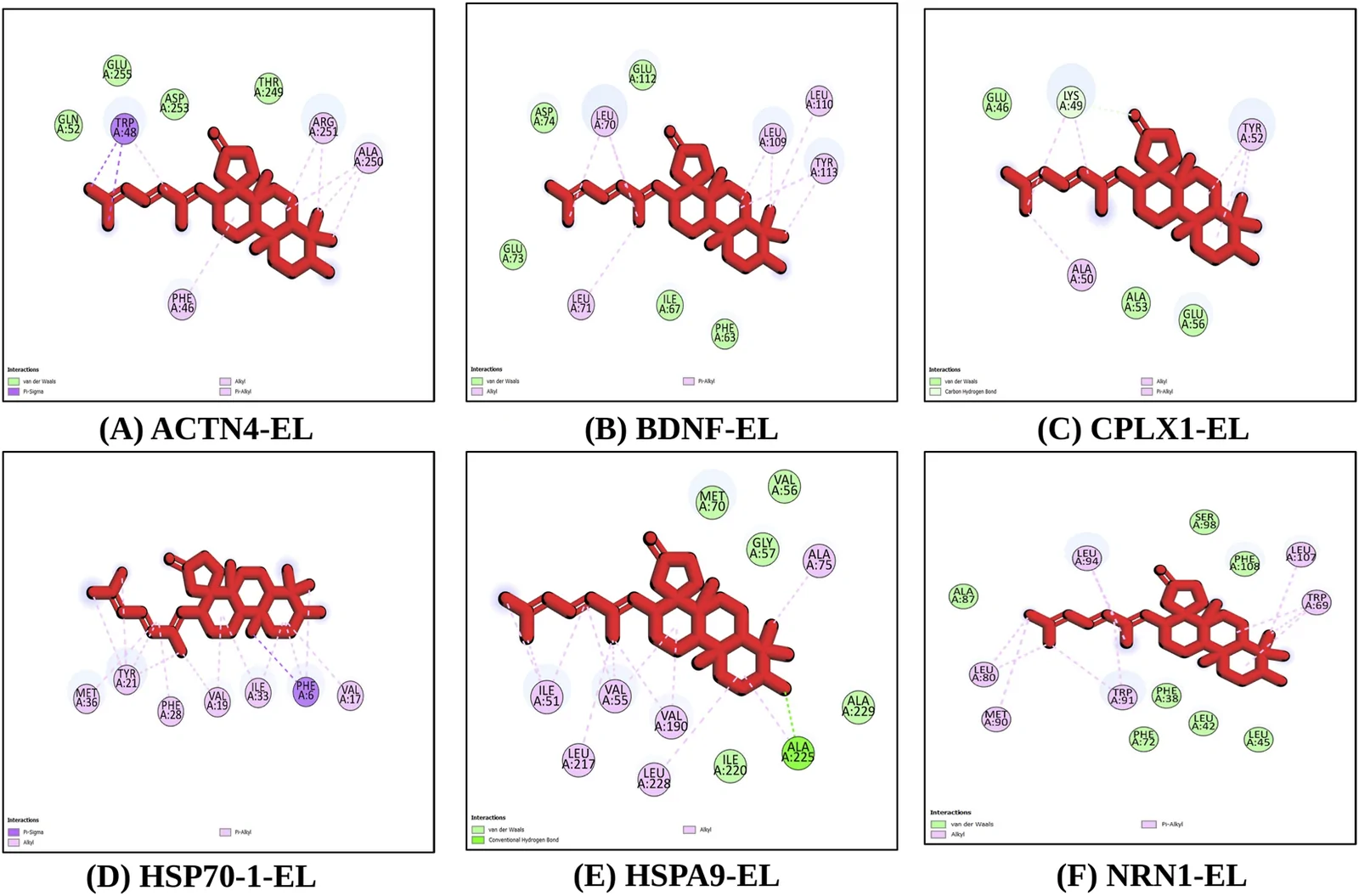
Two-dimensional interaction maps of the selected docked poses of EL with **(A)** ACTN4-EL, **(B)** BDNF-EL, **(C)** CPLX1-EL, **(D)** HSP70-1-EL, **(E)** HSPA9-EL, and **(F)** NRN1-EL.

Across the six complexes, van der Waals and hydrophobic or π-associated contacts were more common than conventional hydrogen bonds. Conventional hydrogen bonds were identified only in the CPLX1–EL and HSPA9–EL poses. This contact pattern was consistent with the rigid triterpenoid framework of EL, its high predicted lipophilicity, and its limited hydrogen-bonding capacity of one donor and three acceptors. The prevalence of nonpolar residues in the predicted binding pockets was also consistent with the dominance of hydrophobic contacts. ACTN4–EL, BDNF–EL, HSP70-1–EL, HSPA9–EL, and NRN1–EL were subsequently selected for molecular dynamics simulations. CPLX1–EL was not advanced to MD because it had the least favorable docking score and occupied a comparatively small, predicted binding pocket. The decision not to advance CPLX1–EL was limited to the MD stage and should not be interpreted as evidence against a potential EL–CPLX1 interaction, because docking results alone cannot establish dynamic stability or functional significance.

### 3.4 Molecular dynamics simulation studies

The 100-ns simulations included five protein–ligand complexes: ACTN4–EL, BDNF–EL, HSP70-1–EL, HSPA9–EL, and NRN1–EL, and their corresponding apo forms. The trajectories were analyzed using protein root-mean-square deviation (RMSD), root-mean-square fluctuation (RMSF), radius of gyration (Rg), and solvent-accessible surface area (SASA), while protein–ligand hydrogen-bond formation was evaluated for the EL-bound complexes. Apo and EL-bound trajectories were compared within each target to examine differences in the sampled protein dynamics.

The RMSD plots show the deviation of the protein from its initial structure during the simulation. Lower RMSD values indicate comparatively lower structural deviation from the starting conformation. The calculated mean RMSD values were ACTN4–EL (0.163 ± 0.021 nm), ACTN4–Apo (0.142 ± 0.023 nm), BDNF–EL (2.125 ± 0.388 nm), BDNF–Apo (1.644 ± 0.182 nm), HSP70-1–EL (0.636 ± 0.088 nm), HSP70-1–Apo (0.801 ± 0.095 nm), HSPA9–EL (0.959 ± 0.152 nm), HSPA9–Apo (1.083 ± 0.119 nm), NRN1–EL (1.141 ± 0.111 nm), and NRN1–Apo (1.676 ± 0.211 nm) (Figure 3; Table 3). Based on the mean RMSD values, the EL-bound forms of HSP70-1, HSPA9, and NRN1 showed lower structural deviation than their corresponding apo forms, whereas ACTN4 and BDNF showed lower RMSD values in the apo forms.

**FIGURE 3:**
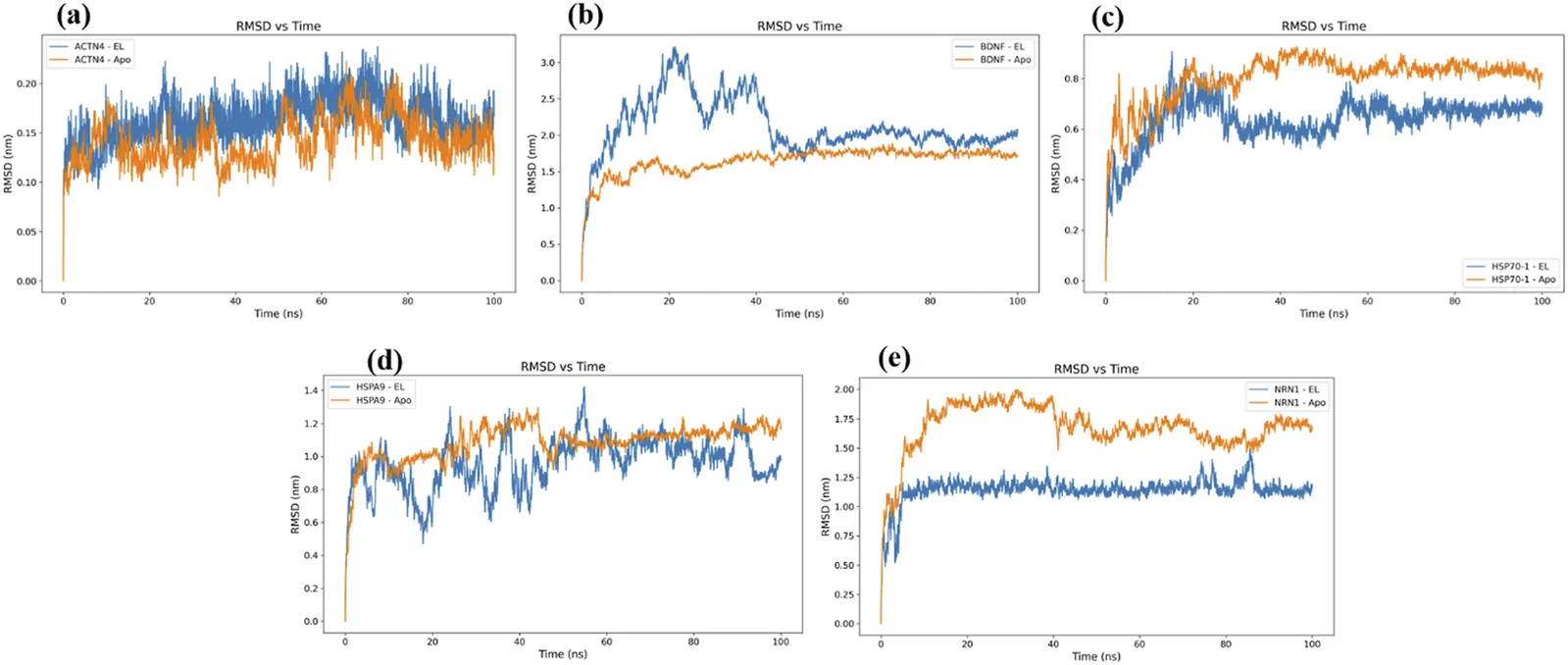
Protein root-mean-square deviation (RMSD) trajectories of **(a)** ACTN4, **(b)** BDNF, **(c)** HSP70-1, **(d)** HSPA9, and **(e)** NRN1 in their EL-bound and corresponding apo forms over 100 ns.

**TABLE 3:** Descriptive trajectory statistics for the EL-bound and corresponding apo forms of ACTN4, BDNF, HSP70-1, HSPA9, and NRN1 over 100 ns.

Complexes	RMSD (nm)	RMSF (nm)	Rg (nm)	SASA (nm^2^)	H-bond
ACTN4 - EL	0.1633 ± 0.0211	0.0928 ± 0.0647	1.8993 ± 0.0109	122.1929 ± 1.9220	0.0936 ± 0.3118
ACTN4 - apo	0.1421 ± 0.0234	0.1006 ± 0.0670	1.8847 ± 0.0117	120.1567 ± 1.9800	-
BDNF - EL	2.1248 ± 0.3878	1.2775 ± 0.8524	2.7117 ± 0.3478	196.2937 ± 11.4141	0.3519 ± 0.5608
BDNF - apo	1.6441 ± 0.1820	0.4336 ± 0.2345	2.2572 ± 0.0883	174.1683 ± 8.5687	-
HSP70-1 - EL	0.6360 ± 0.0882	0.2574 ± 0.1501	1.7972 ± 0.0680	107.8642 ± 3.7876	0.2764 ± 0.6727
HSP70-1 - apo	0.8005 ± 0.0953	0.2781 ± 0.1311	1.7352 ± 0.0439	100.6821 ± 4.1631	-
HSPA9 - EL	0.9591 ± 0.1520	0.4702 ± 0.4320	2.5442 ± 0.1568	221.6653 ± 4.7258	0.1407 ± 0.3735
HSPA9 - apo	1.0829 ± 0.1185	0.2198 ± 0.1957	2.3194 ± 0.0751	206.1569 ± 8.6582	-
NRN1 - EL	1.1406 ± 0.1106	0.3324 ± 0.1903	2.2643 ± 0.0928	112.6760 ± 2.9271	0.5748 ± 0.5006
NRN1 - apo	1.6762 ± 0.2106	0.4399 ± 0.2300	1.8063 ± 0.1363	96.1877 ± 4.9740	-

Values are presented as mean ± standard deviation across trajectory frames. RMSD, root-mean-square deviation; RMSF, root-mean-square fluctuation; Rg, radius of gyration; SASA, solvent-accessible surface area. Protein–ligand hydrogen-bond counts are reported only for the EL-bound complexes.

The RMSF analysis was used to examine residue-level fluctuations during the simulations. The calculated mean RMSF values were ACTN4–EL (0.093 ± 0.065 nm), ACTN4–Apo (0.101 ± 0.067 nm), BDNF–EL (1.278 ± 0.852 nm), BDNF–Apo (0.434 ± 0.235 nm), HSP70-1–EL (0.257 ± 0.150 nm), HSP70-1–Apo (0.278 ± 0.131 nm), HSPA9–EL (0.470 ± 0.432 nm), HSPA9–Apo (0.220 ± 0.196 nm), NRN1–EL (0.332 ± 0.190 nm), and NRN1–Apo (0.440 ± 0.230 nm) (Figure 4; Table 3). Based on the mean RMSF values, ACTN4–EL, HSP70-1–EL, and NRN1–EL showed lower residue-level fluctuations than their corresponding apo forms, whereas BDNF–Apo and HSPA9–Apo showed lower fluctuations than their EL-bound forms.

**FIGURE 4:**
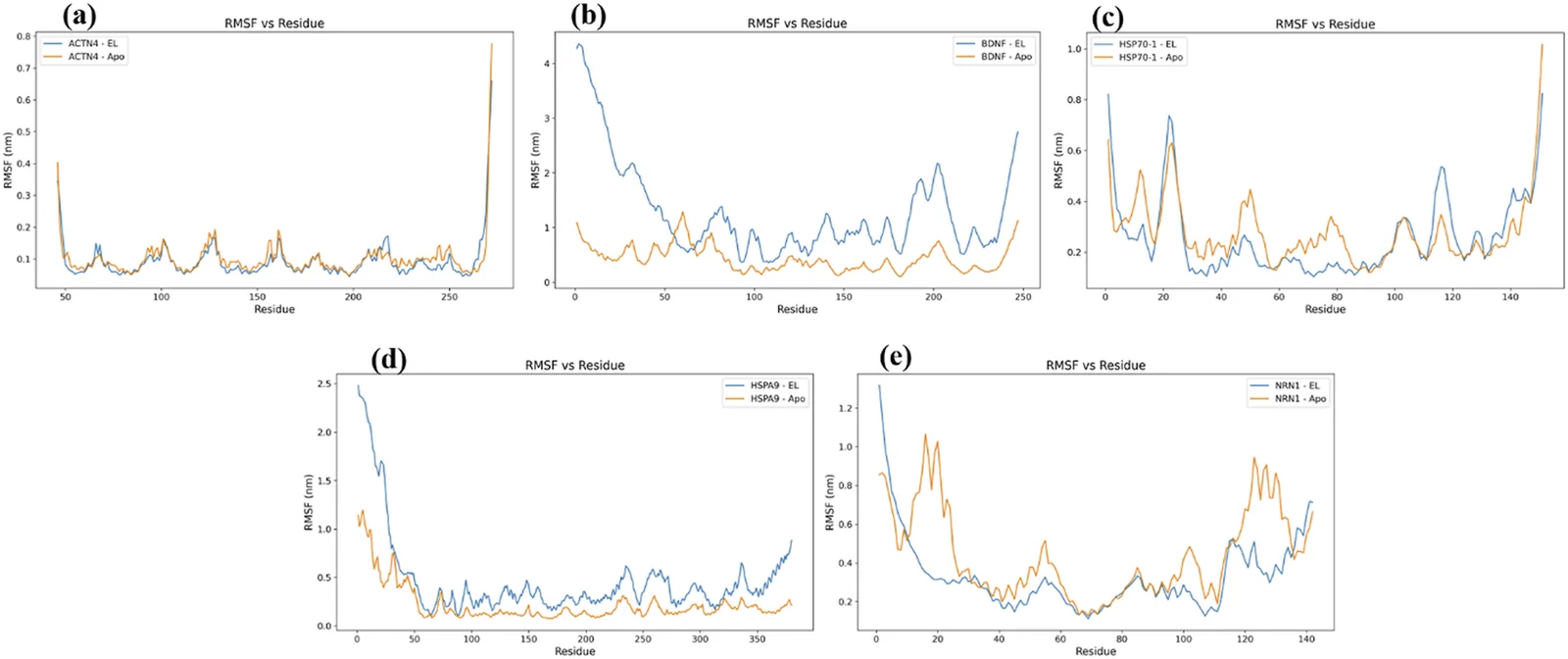
Root-mean-square fluctuation (RMSF) profiles of **(a)** ACTN4, **(b)** BDNF, **(c)** HSP70-1, **(d)** HSPA9, and **(e)** NRN1 in their EL-bound and corresponding apo forms over 100 ns.

The radius of gyration (Rg) analysis was used to examine changes in protein compactness during the simulations. The calculated mean Rg values were ACTN4–EL (1.899 ± 0.011 nm), ACTN4–Apo (1.885 ± 0.012 nm), BDNF–EL (2.712 ± 0.348 nm), BDNF–Apo (2.257 ± 0.088 nm), HSP70-1–EL (1.797 ± 0.068 nm), HSP70-1–Apo (1.735 ± 0.044 nm), HSPA9–EL (2.544 ± 0.157 nm), HSPA9–Apo (2.319 ± 0.075 nm), NRN1–EL (2.264 ± 0.093 nm), and NRN1–Apo (1.806 ± 0.136 nm) (Figure 5; Table 3). Across all five targets, the EL-bound forms showed higher mean Rg values than their corresponding apo forms, consistent with lower average compactness during the EL-bound trajectories.

**FIGURE 5:**
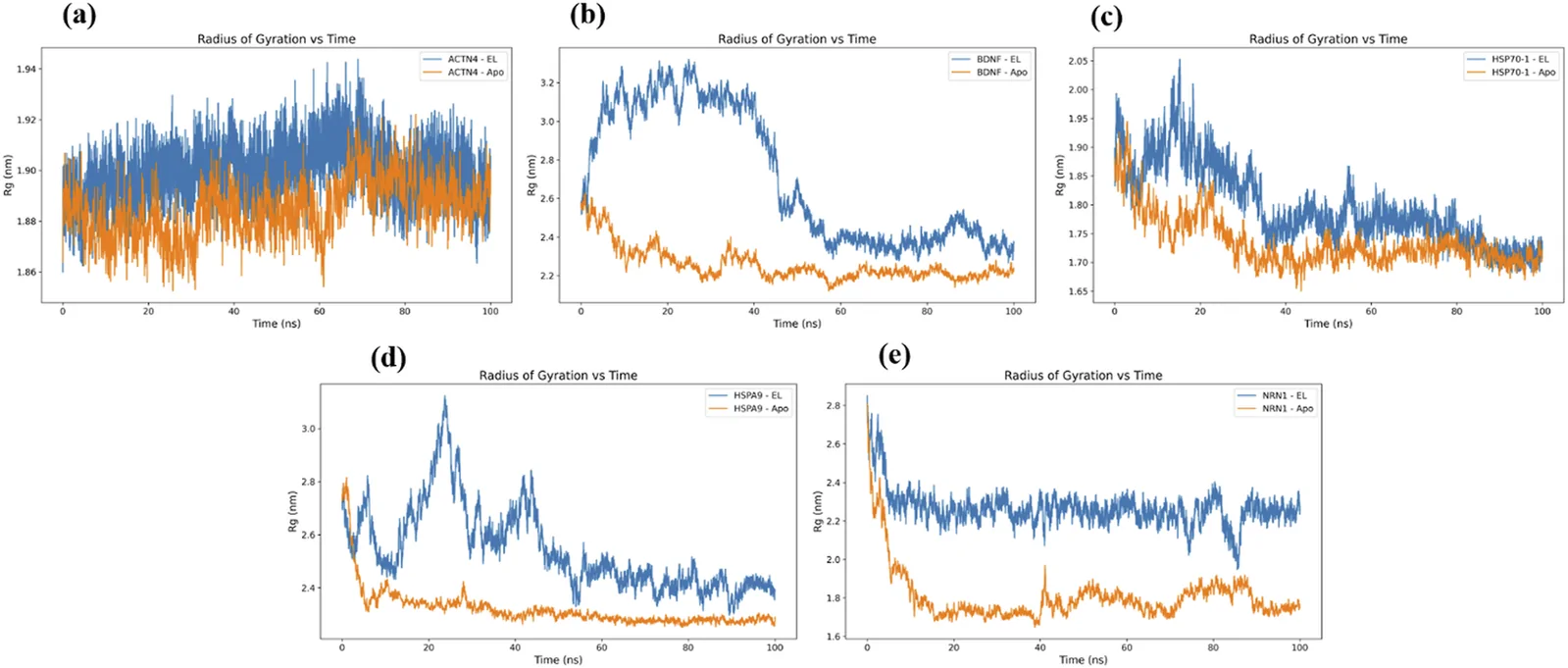
Radius of gyration (Rg) trajectories of **(a)** ACTN4, **(b)** BDNF, **(c)** HSP70-1, **(d)** HSPA9, and **(e)** NRN1 in their EL-bound and corresponding apo forms over 100 ns.

The solvent-accessible surface area (SASA) analysis was used to examine protein surface exposure during the simulations. The calculated mean SASA values were ACTN4–EL (122.193 ± 1.922 nm^2^), ACTN4–Apo (120.157 ± 1.980 nm^2^), BDNF–EL (196.294 ± 11.414 nm^2^), BDNF–Apo (174.168 ± 8.569 nm^2^), HSP70-1–EL (107.864 ± 3.788 nm^2^), HSP70-1–Apo (100.682 ± 4.163 nm^2^), HSPA9–EL (221.665 ± 4.726 nm^2^), HSPA9–Apo (206.157 ± 8.658 nm^2^), NRN1–EL (112.676 ± 2.927 nm^2^), and NRN1–Apo (96.188 ± 4.974 nm^2^) (Figure 6; Table 3). Across all five targets, the EL-bound forms showed higher mean SASA values than their corresponding apo forms, indicating greater average solvent-accessible surface exposure during the EL-bound trajectories. Because the selected protein inputs differed in size and structural coverage, absolute SASA values were interpreted within their respective target comparisons rather than used alone to rank complex stability.

**FIGURE 6:**
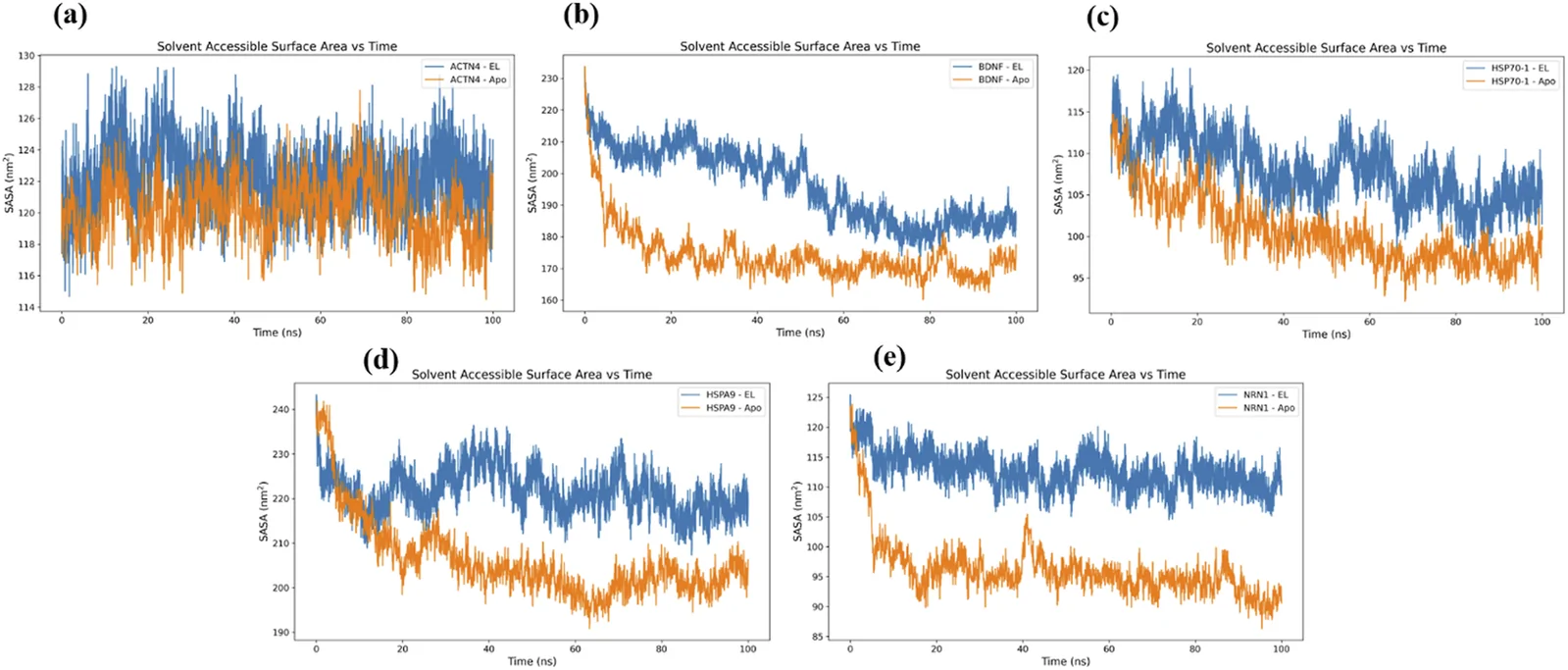
Solvent-accessible surface area (SASA) trajectories of **(a)** ACTN4, **(b)** BDNF, **(c)** HSP70-1, **(d)** HSPA9, and **(e)** NRN1 in their EL-bound and corresponding apo forms over 100 ns.

Protein–ligand hydrogen bonds occurred intermittently across all five EL-bound complexes. The calculated mean hydrogen-bond counts were ACTN4–EL (0.094 ± 0.312), BDNF–EL (0.352 ± 0.561), HSP70-1–EL (0.276 ± 0.673), HSPA9–EL (0.141 ± 0.373), and NRN1–EL (0.575 ± 0.501) (Figure 7; Table 3). NRN1–EL showed the highest mean hydrogen-bond count, followed by BDNF–EL, HSP70-1–EL, HSPA9–EL, and ACTN4–EL. HSP70-1–EL formed multiple simultaneous hydrogen bonds during short intervals, although these were not maintained throughout the trajectory.

**FIGURE 7:**
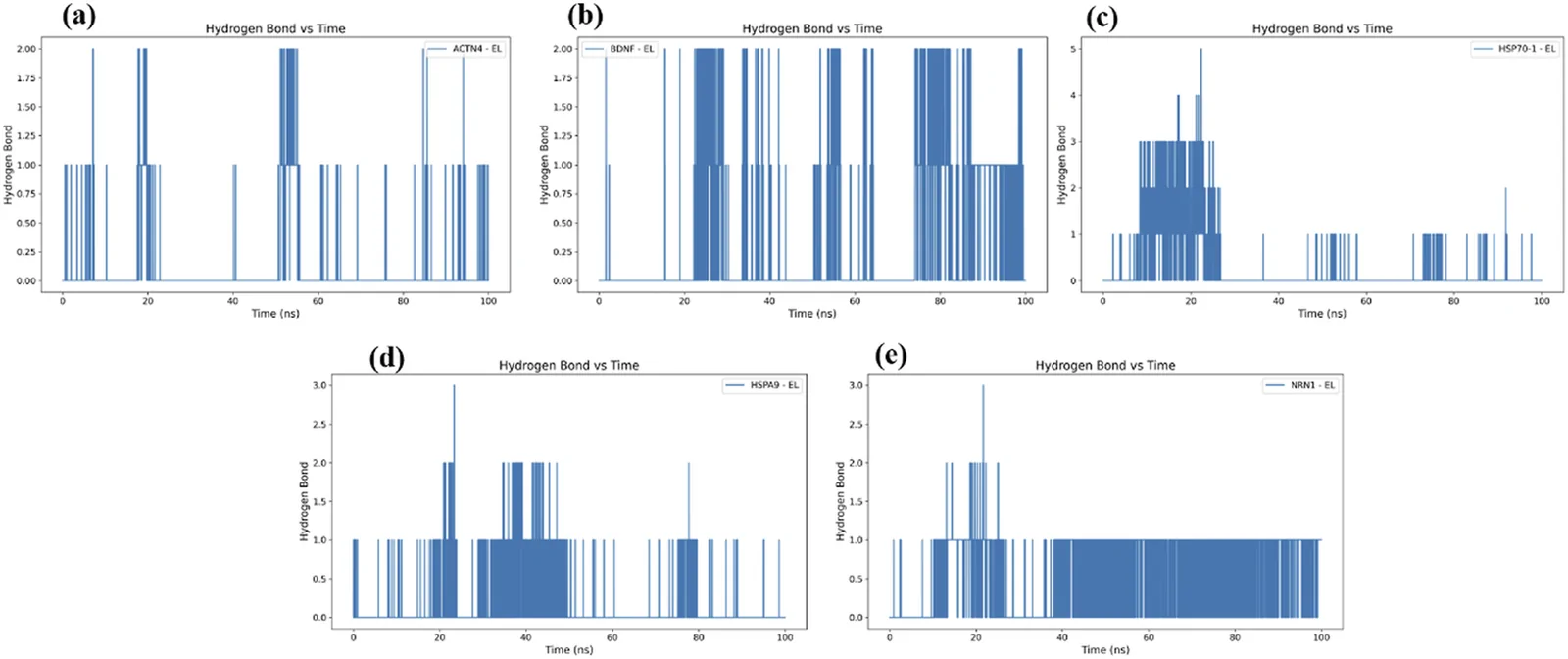
Protein–ligand hydrogen-bond profiles of **(a)** ACTN4–EL, **(b)** BDNF–EL, **(c)** HSP70-1–EL, **(d)** HSPA9–EL, and **(e)** NRN1–EL over 100 ns.

### 3.5 Principal component and free-energy landscape analyses

Principal component analysis and two-dimensional and three-dimensional free-energy landscape plots were examined to understand the conformational states sampled by the EL-bound and corresponding apo forms. The two-dimensional FEL plots are presented in Figure 8, while the corresponding three-dimensional plots are shown in Figure 9. Low-energy regions represent conformational states sampled more frequently during the simulations. Comparison of the apo and EL-bound landscapes showed target-dependent differences in the distribution of sampled conformational states. NRN1–EL displayed a prominent low-energy region, while BDNF–EL and HSPA9–EL also showed identifiable low-energy regions with broader distributions. The NRN1–EL landscape therefore indicated preferential sampling of a dominant conformational region during the simulation. Similar overall patterns were observed in the corresponding three-dimensional FEL plots. These FEL findings were considered together with the RMSD, RMSF, Rg, SASA, hydrogen-bond, and MM/GBSA results when interpreting the conformational behavior of the systems.

**FIGURE 8:**
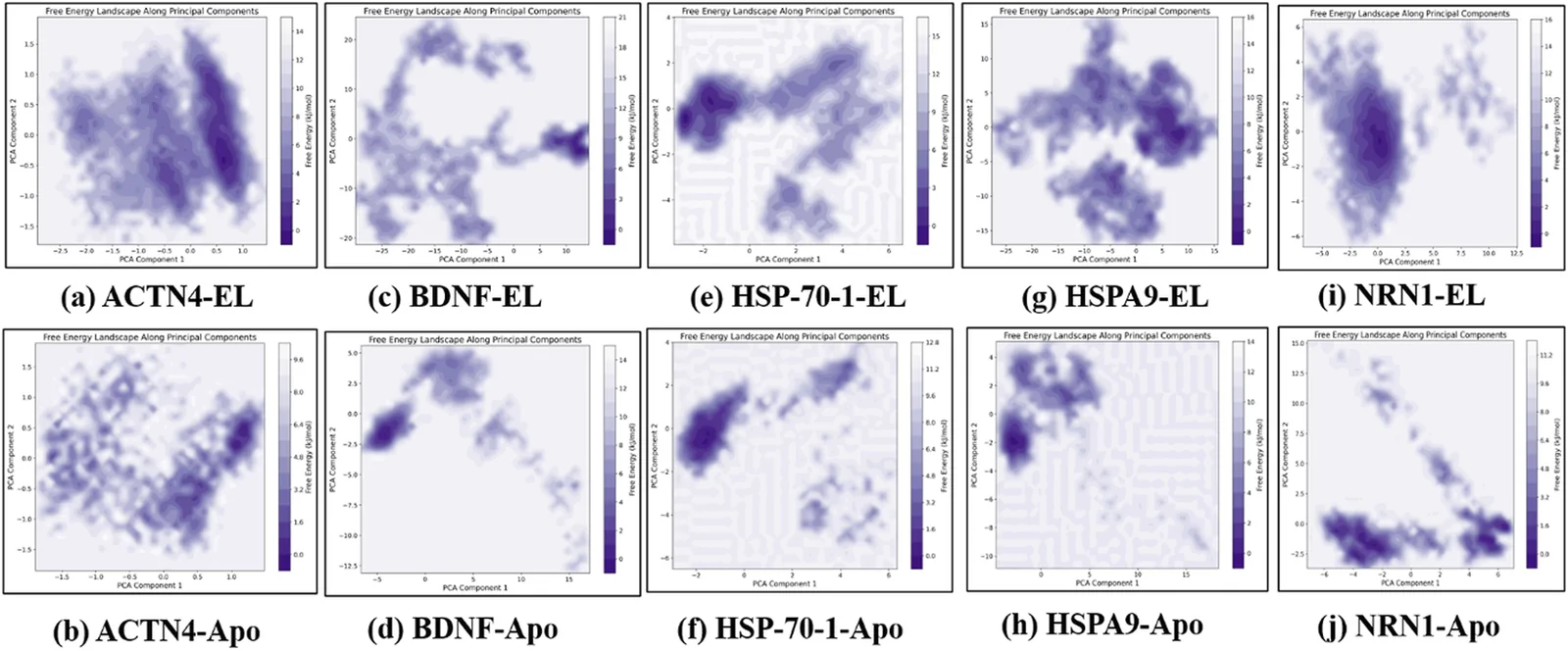
Two-dimensional free-energy landscapes projected onto the first two principal components for **(a)** ACTN4–EL, **(b)** ACTN4–Apo, **(c)** BDNF–EL, **(d)** BDNF–Apo, **(e)** HSP70-1–EL, **(f)** HSP70-1–Apo, **(g)** HSPA9–EL, **(h)** HSPA9–Apo, **(i)** NRN1–EL, and **(j)** NRN1–Apo. Darker blue regions represent lower relative free-energy states and more frequently sampled conformational populations.

**FIGURE 9:**
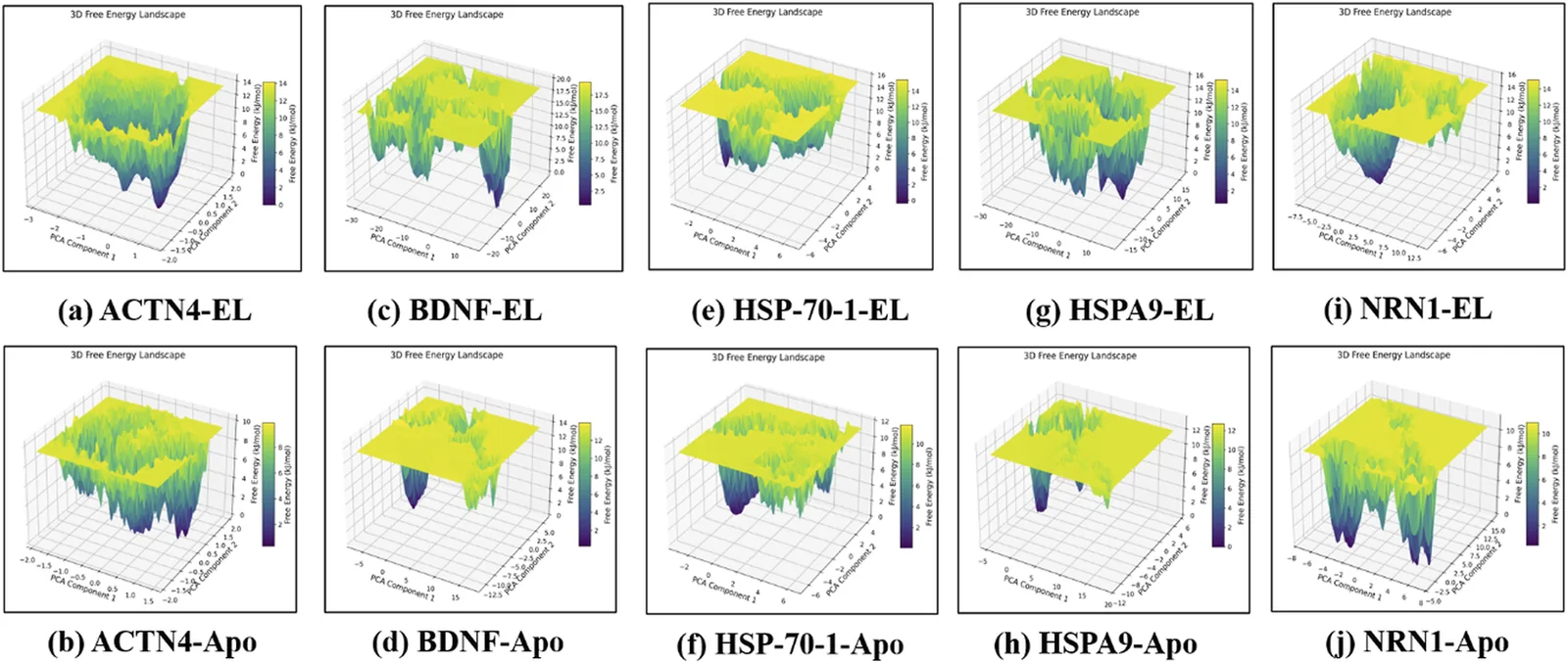
Three-dimensional free-energy landscapes for **(a)** ACTN4–EL, **(b)** ACTN4–Apo, **(c)** BDNF–EL, **(d)** BDNF–Apo, **(e)** HSP70-1–EL, **(f)** HSP70-1–Apo, **(g)** HSPA9–EL, **(h)** HSPA9–Apo, **(i)** NRN1–EL, and **(j)** NRN1–Apo. Valleys represent regions of lower relative free energy and more frequently sampled conformational populations.

### 3.6 MM/GBSA binding-energy estimates and per-residue energy decomposition

MM/GBSA calculations were performed using gmx_MMPBSA to estimate the binding energies of the five simulated protein–ligand complexes. The analysis included van der Waals energy (ΔVDWAALS), electrostatic energy (ΔEEL), generalized Born polar-solvation energy (ΔEGB), nonpolar solvation energy (ΔESURF), gas-phase energy (ΔGGAS), total solvation energy (ΔGSOLV), and total binding energy (ΔTOTAL). The calculated energy components are presented in Figure 10. The mean total MM/GBSA binding-energy estimates were ACTN4–EL (−8.03 ± 2.15 kcal mol^−1^), BDNF–EL (−33.83 ± 3.49 kcal mol^−1^), HSP70-1–EL (−23.20 ± 5.24 kcal mol^−1^), HSPA9–EL (−37.93 ± 2.60 kcal mol^−1^), and NRN1–EL (−39.21 ± 3.24 kcal mol^−1^). NRN1–EL and HSPA9–EL showed the two most negative mean ΔTOTAL estimates, followed by BDNF–EL, HSP70-1–EL, and ACTN4–EL. Because entropic contributions were not included and the calculations were derived from single MD trajectories, these values were interpreted as comparative computational estimates rather than absolute experimental binding free energies. The relatively similar estimates for NRN1–EL and HSPA9–EL were therefore not used to establish a rigorous quantitative ranking between these two complexes.

**FIGURE 10:**
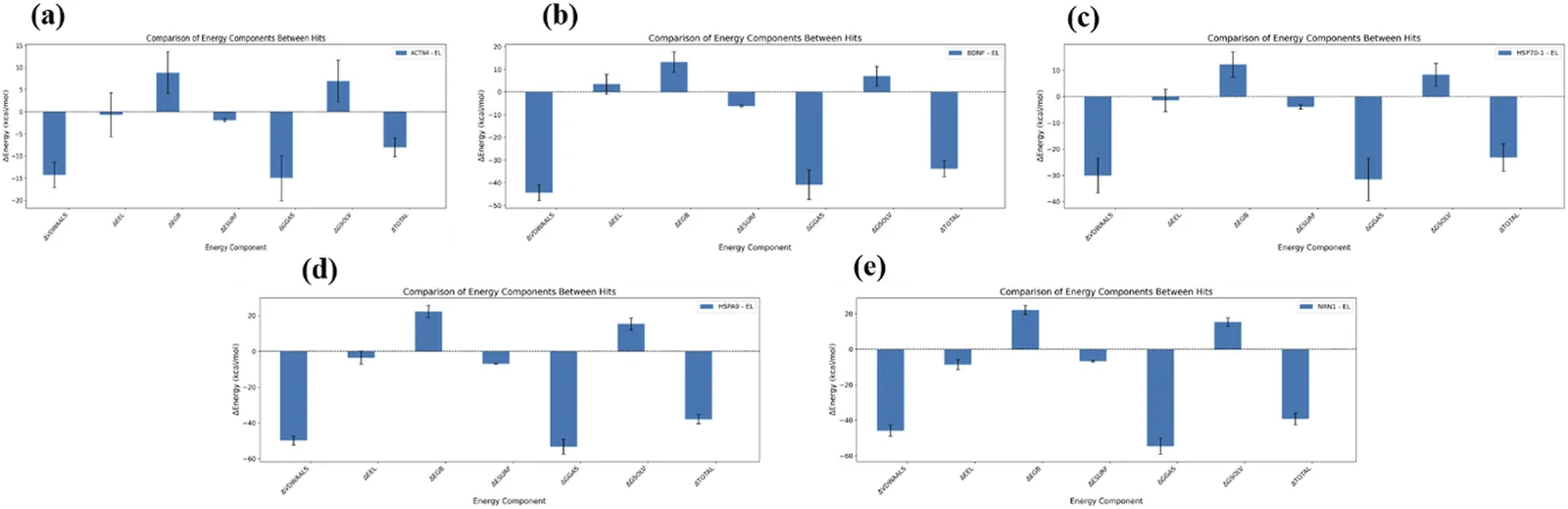
MM/GBSA binding-energy components for **(a)** ACTN4–EL, **(b)** BDNF–EL, **(c)** HSP70-1–EL, **(d)** HSPA9–EL, and **(e)** NRN1–EL.

Per-residue energy decomposition was additionally performed to examine residue-level contributions to the calculated MM/GBSA energies (Figure 11). For ACTN4–EL, the protein-side residue contributions were comparatively small at the scale of the decomposition plot. In the other complexes, more pronounced favorable contributions were observed for several residues, including LEU70, LEU109, and TYR113 in BDNF–EL; VAL19, TYR21, ILE33, and MET36 in HSP70-1–EL; MET70, VAL190, ALA225, and LEU228 in HSPA9–EL; and PHE38, LEU94, LEU107, and PHE108 in NRN1–EL. These residue-level profiles provide additional information on the predicted energetic contributions within each complex but do not independently establish experimental binding-site relevance.

**FIGURE 11:**
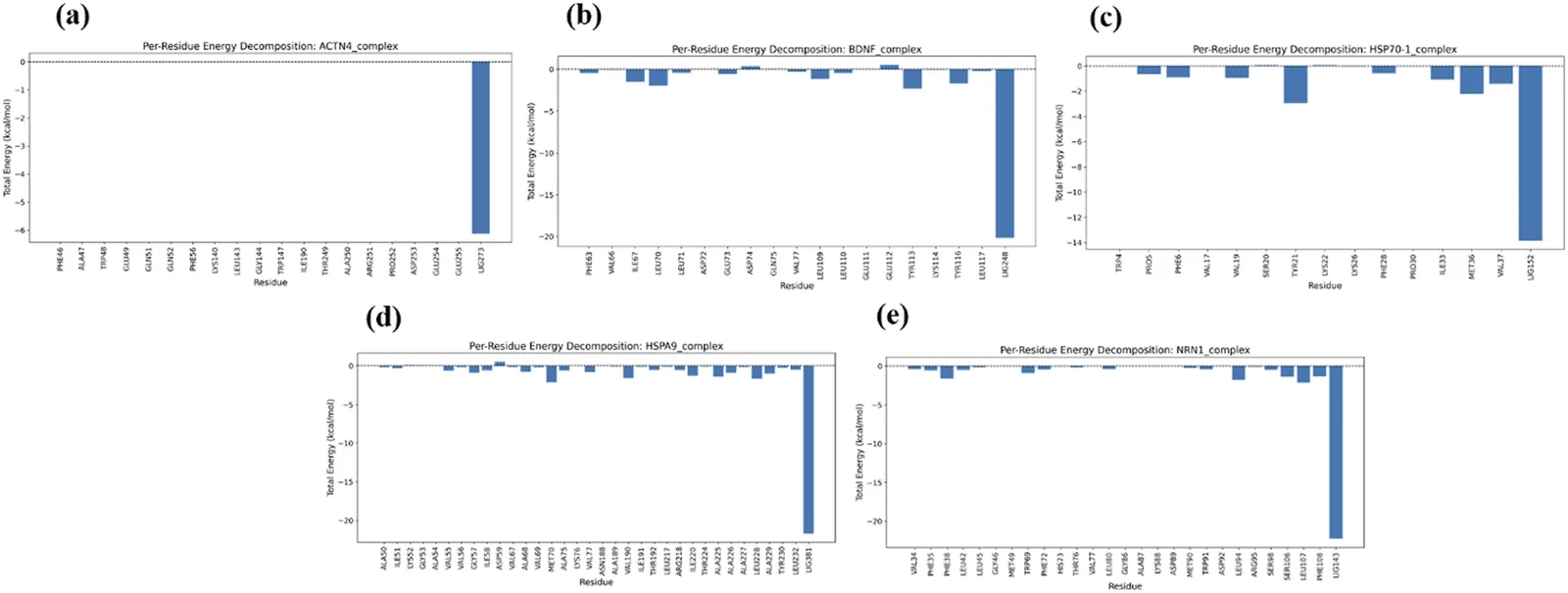
Per-residue energy decomposition profiles for **(a)** ACTN4–EL, **(b)** BDNF–EL, **(c)** HSP70-1–EL, **(d)** HSPA9–EL, and **(e)** NRN1–EL.

## 4 Discussion

The ADMET profile of ebelin lactone (EL) and its predicted interactions with six cognition-associated proteins were analyzed using molecular docking, 100-ns molecular dynamics simulations of both EL-bound and corresponding apo forms, principal component and free-energy landscape analyses, and MM/GBSA binding-energy estimation with per-residue energy decomposition. The different computational analyses did not produce an identical ranking across the selected targets. HSPA9–EL showed the most favorable docking score, whereas NRN1–EL showed the most negative MM/GBSA binding-energy estimate, the highest mean protein–ligand hydrogen-bond count, and a prominent low-energy region in the free-energy landscape. NRN1–EL also showed lower mean RMSD and RMSF than apo NRN1, although its Rg and SASA values were higher. Considering the results together, NRN1 was prioritized as the overall most promising computational candidate for experimental follow-up. Although HSPA9 showed the most favorable docking score and a similarly favorable MM/GBSA estimate, the close MM/GBSA values were not used alone to rank the two complexes.

NRN1 has been associated with cognitive resilience and neuronal plasticity (Hurst et al., 2023), while HSPA9 is a mitochondrial chaperone involved in protein folding and cellular stress responses (Flachbartová and Kovacech, 2013). However, the predicted EL–NRN1 and EL–HSPA9 interactions do not establish modulation of these biological functions and require experimental confirmation. The ADMET predictions provided additional information on the developability of EL. EL showed high predicted lipophilicity and limited aqueous solubility, which may create challenges for formulation and bioavailability (Bunally et al., 2019; Gallego et al., 2024). Although some permeability-related outputs were numerically high, the available predictions were not sufficient to make definitive conclusions regarding oral bioavailability, CNS exposure, or safety. Further experimental assessment of solubility, permeability, systemic exposure, and safety would therefore be required.

Several limitations define the scope of the present findings. The protein inputs differed in structural source and sequence coverage, which limits direct comparison of absolute docking and trajectory measures across the targets. The docking poses were generated within predicted binding regions without experimental binding-site validation or redocking controls. CPLX1–EL was not advanced to molecular dynamics; therefore, its dynamic behavior remains unknown. The corresponding apo simulations provided a reference for interpreting the EL-bound trajectories; however, each system was represented by a single 100-ns trajectory without independent simulation replicates. The MM/GBSA calculations excluded entropic contributions and remain sensitive to conformational sampling and the structural models used. Similarly, the per-residue energy decomposition represents predicted energetic contributions and does not provide experimental evidence of residue-level binding. These limitations should be considered when interpreting the target prioritization. Experimental binding studies and functional cellular assays are required to validate the predicted EL–NRN1 and EL–HSPA9 interactions and determine their biological relevance.

## 5 Conclusion

Integration of the docking, 100-ns molecular dynamics, free-energy landscape, and MM/GBSA results prioritized NRN1–EL for experimental follow-up among the complexes examined. Based on the integrated findings, NRN1 emerged as the overall most promising computational candidate, showing the most negative MM/GBSA estimate, the highest mean hydrogen-bond count, and a prominent low-energy conformational region. HSPA9 showed the most favorable docking score and a similarly favorable MM/GBSA estimate; however, these findings were not sufficient to prioritize HSPA9 over NRN1 when the results were considered together. Comparison with the corresponding apo trajectories showed target-dependent changes, with NRN1–EL exhibiting lower mean RMSD and RMSF than apo NRN1, although its Rg and SASA values were higher. These findings support further experimental evaluation of the predicted EL–NRN1 interaction and should be interpreted as a hypothesis-generating computational prediction until experimentally validated.

## Data Availability

The raw data supporting the conclusions of this article will be made available by the authors, without undue reservation.

## Footnotes

1 ScientiFlow. Available at: https://scientiflow.com/
