# Supplementary material for: Comparative multi-target computational profiling of ebelin lactone across cognition-associated proteins
Source: Front Bioinform. 2026 Oct 23;6:1952759. doi: 10.3389/fbinf.2026.1952759 (PMC13739814; doi:10.3389/fbinf.2026.1952759)
Supplement: Supplementary file 1 [file Table1.docx]

**Table S1.**  ADMET predictions of Ebelin Lactone (EL)

| ADMET endpoint | Predicted output |
| --- | --- |
| SMILES | CC(=C/C=C/C(=C/[C@H]1CC[C@@H]2[C@]3(CC[C@@H](C([C@@H]3CC[C@]2([C@]14CC(=O)OC4)C)(C)C)O)C)/C)C |
| Molecular weight | 454.695 |
| logP | 7.0181 |
| Hydrogen bond acceptors | 3 |
| Hydrogen bond donors | 1 |
| Lipinski | 3 |
| QED | 0.36811 |
| Stereo centers | 7 |
| TPSA | 46.53 |
| AMES | 0.225214 |
| BBB Martins | 0.206425 |
| Bioavailability Ma | 0.44261 |
| CYP1A2 Veith | 0.004031 |
| CYP2C19 Veith | 0.036605 |
| CYP2C9 Substrate CarbonMangels | 0.01803 |
| CYP2C9 Veith | 0.083377 |
| CYP2D6 Substrate CarbonMangels | 0.021727 |
| CYP2D6 Veith | 0.005579 |
| CYP3A4 Substrate CarbonMangels | 0.745342 |
| CYP3A4 Veith | 0.360044 |
| Carcinogens Lagunin | 0.074403 |
| ClinTox | 0.084488 |
| DILI | 0.204676 |
| HIA Hou | 0.992545 |
| NR-AR-LBD | 0.364572 |
| NR-AR | 0.272554 |
| NR-AhR | 0.002601 |
| NR-Aromatase | 0.490033 |
| NR-ER-LBD | 0.356381 |
| NR-ER | 0.444586 |
| NR-PPAR-gamma | 0.134795 |
| PAMPA NCATS | 0.925511 |
| Pgp Broccatelli | 0.821793 |
| SR-ARE | 0.826617 |
| SR-ATAD5 | 0.085712 |
| SR-HSE | 0.410632 |
| SR-MMP | 0.590318 |
| SR-p53 | 0.571862 |
| Skin Reaction | 0.757241 |
| hERG | 0.505042 |
| Caco2 Wang | -5.23412 |
| Clearance Hepatocyte AZ | 76.68233 |
| Clearance Microsome AZ | 53.0684 |
| Half Life Obach | 7.659937 |
| HydrationFreeEnergy FreeSolv | -4.05964 |
| LD50 Zhu | 3.319639 |
| Lipophilicity AstraZeneca | 4.043488 |
| PPBR AZ | 101.324 |
| Solubility AqSolDB | -6.79951 |
| VDss Lombardo | -7.6661 |
| molecular weight drugbank approved percentile | 76.19232 |
| logP drugbank approved percentile | 97.20822 |
| hydrogen bond acceptors drugbank approved percentile | 31.17487 |
| hydrogen bond donors drugbank approved percentile | 36.68088 |
| Lipinski drugbank approved percentile | 20.91896 |
| QED drugbank approved percentile | 29.97286 |
| stereo centers drugbank approved percentile | 89.78286 |
| tpsa drugbank approved percentile | 28.26677 |
| AMES drugbank approved percentile | 56.22334 |
| BBB Martins drugbank approved percentile | 12.91198 |
| Bioavailability Ma drugbank approved percentile | 15.97518 |
| CYP1A2 Veith drugbank approved percentile | 23.22606 |
| CYP2C19 Veith drugbank approved percentile | 32.88096 |
| CYP2C9 Substrate CarbonMangels drugbank approved percentile | 6.281504 |
| CYP2C9 Veith drugbank approved percentile | 62.38852 |
| CYP2D6 Substrate CarbonMangels drugbank approved percentile | 15.47111 |
| CYP2D6 Veith drugbank approved percentile | 18.57309 |
| CYP3A4 Substrate CarbonMangels drugbank approved percentile | 79.7596 |
| CYP3A4 Veith drugbank approved percentile | 74.56378 |
| Carcinogens Lagunin drugbank approved percentile | 29.31369 |
| ClinTox drugbank approved percentile | 47.38271 |
| DILI drugbank approved percentile | 33.34626 |
| HIA Hou drugbank approved percentile | 44.55215 |
| NR-AR-LBD drugbank approved percentile | 94.30012 |
| NR-AR drugbank approved percentile | 93.5634 |
| NR-AhR drugbank approved percentile | 17.29352 |
| NR-Aromatase drugbank approved percentile | 96.7817 |
| NR-ER-LBD drugbank approved percentile | 96.23885 |
| NR-ER drugbank approved percentile | 93.95114 |
| NR-PPAR-gamma drugbank approved percentile | 93.13687 |
| PAMPA NCATS drugbank approved percentile | 72.62505 |
| Pgp Broccatelli drugbank approved percentile | 88.83288 |
| SR-ARE drugbank approved percentile | 96.4715 |
| SR-ATAD5 drugbank approved percentile | 86.46762 |
| SR-HSE drugbank approved percentile | 96.23885 |
| SR-MMP drugbank approved percentile | 88.87166 |
| SR-p53 drugbank approved percentile | 97.20822 |
| Skin Reaction drugbank approved percentile | 80.10857 |
| hERG drugbank approved percentile | 64.05584 |
| Caco2 Wang drugbank approved percentile | 34.93602 |
| Clearance Hepatocyte AZ drugbank approved percentile | 80.96161 |
| Clearance Microsome AZ drugbank approved percentile | 79.1392 |
| Half Life Obach drugbank approved percentile | 50.87243 |
| HydrationFreeEnergy FreeSolv drug bank approved percentile | 92.43893 |
| LD50 Zhu drugbank approved percentile | 89.22063 |
| Lipophilicity AstraZeneca drugbank approved percentile | 95.73478 |
| PPBR AZ drugbank approved percentile | 92.74913 |
| Solubility AqSolDB drugbank approved percentile | 1.822412 |
| VDss Lombardo drugbank approved percentile | 3.257076 |

**Note:** ADMET-AI outputs are presented as generated by the platform and include classification probabilities, regression estimates, and DrugBank-approved percentile values. Values without a specified threshold or class direction were not assigned positive or negative classifications.
